# Childhood Maltreatment and Cardiovascular Health: Role of Sex and Life’s Essential 8 in the U.K. Biobank

**DOI:** 10.1016/j.jacadv.2025.102483

**Published:** 2026-01-28

**Authors:** Bikram Bucha, Yuanjing Li, Örjan Ekblom, Victoria Blom, Xin Xia, Hui-Xin Wang, Rui Wang

**Affiliations:** a Department of Physical Activity and Health, the Swedish School of Sport and Health Science, GIH, Stockholm, Sweden; b Division of Nursing, Department of Neurobiology, Care Sciences and Society, Karolinska Institutet, Stockholm, Sweden; c Division of Neurogeriatrics, Department of NVS, Karolinska Institutet, Stockholm, Sweden; d Stress Research Institute, Department of Psychology, Stockholm University, Stockholm, Sweden; e Department of Clinical Neuroscience, Karolinska Institutet, Stockholm, Sweden; f Division of Clinical Geriatrics, Department of Neurobiology, Care Sciences and Society, Karolinska Institutet, Stockholm, Sweden; g Wisconsin Alzheimer’s Disease Research Center, University of Wisconsin School of Medicine and Public Health, Madison, Wisconsin, USA

**Keywords:** cardiovascular disease, cardiovascular health, Life’s essential 8, childhood maltreatment, childhood adversity

## Abstract

**Background:**

Childhood maltreatment (CM) may increase cardiovascular disease (CVD) risk through behavioral and health pathways; while maintaining cardiovascular health, Life’s Essential 8 (LE8) may modify this association.

**Objectives:**

The objective of the study was to examine the link between CM and LE8, and to quantify the potential moderating role of LE8 scores in CM-related CVD risk for men and women.

**Methods:**

We included 153,399 participants aged ≥40 years at baseline in the U.K. Biobank. CM was assessed using the online Childhood Trauma Screener. LE8 scores were calculated based on 8 behavioral and health factors. Incident CVDs were identified through linkage to patient and death registers up to 2022. Linear and Cox regressions were used.

**Results:**

All CM events were significantly associated with lower LE8 scores in both sexes, with stronger associations observed with increasing cumulative CM exposure (β_1CM_ = −0.58, 95% CI: −0.72 to −0.44; β_2CM_ = −1.17, 95% CI: −1.36 to −0.97; β_≥3CM_ = −2.26, 95% CI: −2.47 to −2.05). Compared to individuals with no CM exposure and low LE8 scores, those maintaining moderate-to-high LE8 scores demonstrated a substantial reduction in risk for all CVD events, regardless of the number of CM events experienced. However, among participants with low total LE8 scores, women appeared more vulnerable than men to myocardial infarction when exposed to cumulative CM events.

**Conclusions:**

The impact of early-life adversities can be offset by adopting good lifelong lifestyle practices. This study underscores the need to identify individuals, particularly women, with experiences of cumulative CM events and support interventions to improve their opportunity, capability, and motivation to enhance LE8.

Childhood maltreatment (CM), a form of adverse childhood experience, includes several forms of abuse and neglect occurring before the age of 18.^1^ CM has been linked to the development of cardiovascular disease (CVD) later in life.^2^ For instance, childhood abuse has been associated with lifelong psychological and behavioral disruptions, which in turn increase the risk of CVD in adulthood.^3^ Experiencing neglect during childhood, particularly emotional neglect, has been connected to adult CVD, potentially through pathways involving chronic stress, unhealthy behaviors, and inflammation.^4^ Despite consistent evidence from previous research, effective strategies for CVD prevention in individuals with a history of CM remain unclear.^5^^,^^6^

In 2022, the American Heart Association (AHA) introduced the Life’s Essential 8 (LE8) guidelines to promote cardiovascular health by encouraging healthy behaviors and maintaining optimal levels of 4 cardiometabolic health factors (eg, blood pressure, body mass index [BMI]).^7^ Extensive evidence from large cohort studies, has demonstrated that keeping an optimal LE8 level is prominently associated with reduced CVD risk and mortality.^8^ However, the prevalence of adults meeting this optimal LE8 level is reportedly low,^9^ with just 19.6% in the United States.^10^ Investigating the role of LE8 in the link between CM and CVD thus becomes critical for several reasons. First, most existing research has focused on single cardiovascular factors, highlighting the need to examine how CM and its subtypes relate to overall LE8 scores, including both behavioral and cardiometabolic components.^11^^,^^12^ Second, maintaining an optimal LE8 scores, particularly its behavioral component, reflects personal choices. Understanding how CM relates to the LE8 components may reveal key pathways linking CM to CVD, thereby informing the development of targeted, personalized intervention strategies. Third, there is currently no evidence on the extent to which maintaining optimal LE8 levels in adulthood can mitigate the harmful effects of CM on CVD. Addressing such a protective effect would support the applicability of the AHA guidelines for at-risk populations.

Furthermore, sex differences are another important factor in the relationship between CM and cardiovascular health.^13^ Women may exhibit greater hormonal influences (eg, myocardial remodeling under stress and drug metabolism via sex-specific enzymes), whereas men are more easily influenced by unhealthy behaviors.^13^ In this context, women are likely to exhibit stronger associations between CM and the cardiometabolic components of LE8, whereas men tend to show stronger links to the behavioral components.^14^^,^^15^ However, evidence on sex-specific patterns linking CM to LE8 levels, particularly its subcomponents, remains limited.^15^^,^^16^ It is still unknown whether men and women benefit differently from optimal maintenance of LE8 in mitigating the impact of CM on CVD.

Using data from the U.K. Biobank cohort, we aimed to: 1) examine the overall and sex-specific association between CM and LE8 scores, including its subcomponents; and 2) assess whether maintaining an optimal LE8 score can mitigate the adverse associations between CM and specific CVD outcomes, and whether these associations vary by sex. The study specifically focused on both acute and chronic CVD events involving the heart (ie, myocardial infarction [MI]), congestive heart failure [CHF]), blood vessels (peripheral vascular disease), and brain (cerebrovascular disease).

## Methods

### Study design and study participants

This study used data from the U.K. Biobank Cohort (https://www.ukbiobank.ac.uk/), a large prospective study that recruited over 500,000 participants aged ≥40 years across the United Kingdom between 2006 and 2010. The study received ethical approval from the U.K. Biobank Resource (application number 86931) and the Swedish Ethical Review Authority (2024-00716-01). Information on the selection of study participants is provided in the flowchart (Supplemental Figure 1). Specifically, of the 502,382 baseline participants, 157,253 completed an online questionnaire to recall the history of CM in 2016. After excluding 3,711 participants who responded “prefer not to say” to the questionnaires and 143 with missing LE8 scores, 153,399 individuals were included in our analytical sample, focusing on the association between CM and baseline LE8 scores (aim 1) (Central Illustration). Similarly, among participants who responded to CM questionnaires, 23,610 were excluded owing to CVD events before baseline (4,352), responded “prefer not to say” (3,296), withdrew, or had no follow-up information (15,962). Finally, 133,643 individuals were included in our analytical sample investigating the association between CM, LE8 scores, and incident CVD events (aim 2).

**Central Illustration:**
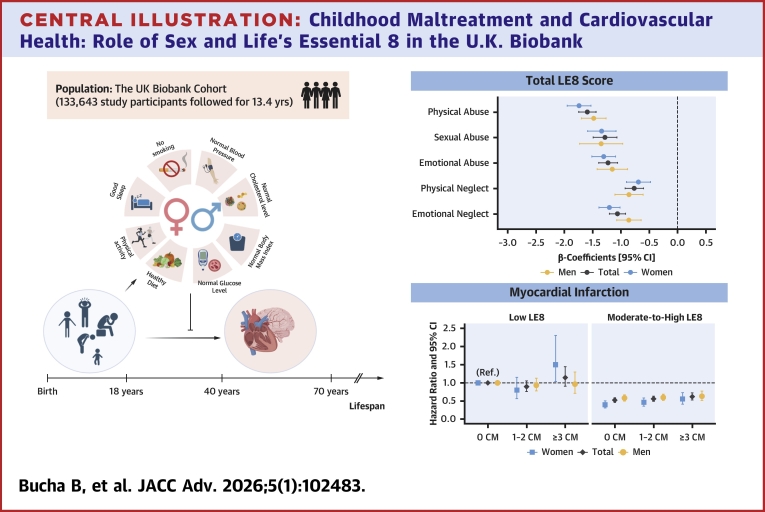
**Childhood Maltreatment and Cardiovascular Health: Role of Sex and Life’s Essential 8 in the U.K. Biobank** Multivariable linear regression models were applied to estimate the beta-coefficients for the association between childhood maltreatment events and total LE8 scores. Cox-proportional hazard regression models were applied to estimate the association between CM numbers and incident CVD events by LE8 levels and sex. All models were adjusted for age, sex, ethnicity, education, Townsend Deprivation Index, and Charlson Comorbidity Index. CM = childhood maltreatment; LE8 = Life’s Essential 8.

### Assessment of childhood maltreatment

CM was assessed using a validated and reliable childhood trauma screener (CTS).^17^ The CTS is a five-point Likert scale item for specific CM events: physical abuse, physical neglect, sexual abuse, emotional abuse, and emotional neglect.^17^ The details of the assessment and cutoffs for each CM event are shown in Supplemental Table 1. We also calculated a total score by summing CM events, ranging from 0 to 5, with 0 indicating no experience and higher scores reflecting greater cumulative exposure. The total score was categorized as none, 1, 2, or 3 or more events for analysis.^18^

### Assessment of LE8

The LE8 score was calculated based on AHA’s guidelines, with modifications to diet scores due to data availability.^19^ It comprises 8 metrics, including health behaviors and health factors.^7^ The behavioral component included self-reported data on diet, physical activity, smoking, and sleep, whereas the health factors consisted of BMI, blood glucose, cholesterol, and blood pressure measured at assessment centers.^7^ Each metric was scored from 0 to 100, with higher scores indicating better health.^7^ Detailed scoring criteria are provided in Supplemental Tables 2 and 3. The average scores for the 2 components and total LE8, ranging from 0 to 100, were categorized as low (0-49), moderate (50-79), or high (80-100), per AHA’s recommendations.^7^ Since reaching a high LE8 level can be challenging for the general population, we examined its modifying effect when individuals met at least the minimum requirement, using a binary variable: moderate-to-high vs low LE8 levels.

### Incident acute and chronic CVD events

CVD events were identified using the International Classification of Diseases-10th Revision (ICD-10), through linkage to patient and death registers up to December 2022. The 4 CVD events were classified as follows: MI (ICD-10 codes: I21, I22, I252), cerebrovascular disease (ICD-10 codes: I60-I69, G45, G46), CHF (ICD-10 codes: I110, I130, I132, I50), and peripheral vascular disease (ICD-10 codes: I70, I71, I731, I738, I739, I771, I790, I792, K551, K558, K559, R02, Z958, Z959).

### Demographic factors and other covariates

The baseline age of study participants was calculated. Sex was self-reported and categorized as men vs women. Ethnicity was classified as White vs non-White. Education level was based on participants’ highest qualification, categorized into low, medium, or high.^20^ Socioeconomic deprivation was assessed using the validated Townsend Deprivation Index (TDI), with higher scores or higher quartiles indicating higher levels of deprivation.^21^ Baseline somatic health was estimated using the Charlson Comorbidity Index (CCI), which summarizes 19 medical conditions based on U.K. Biobank Inpatient data using ICD-10 codes (Supplemental Table 4).^22^ In the analysis involving CVD outcomes, we excluded relevant events from the original CCI in the model.

### Statistical analysis

Baseline characteristics of study participants were compared by sex using a t-test for continuous variables and a chi-square test for categorical variables. Linear regression models examined the relationship linking individual CM events and their accumulation to LE8 scores. Sex-specific associations were assessed through stratified analyses for men and women and by introducing interaction terms between sex and CM events in the models. To evaluate the modifying role of maintaining an optimal LE8 level in the association between CM and CVD risk, we first used Cox proportional hazards regression to assess the association between CM and specific CVD events. Analyses were further stratified by sex and LE8 levels to explore the sex-specific associations. The proportional-hazards assumption was tested using Schoenfeld (scaled) residuals and a global test, whereas collinearity was assessed using variance inflation factors. Models were adjusted for demographic factors (baseline age, sex, ethnicity, education, and TDI) and baseline somatic health to control for covariates that may influence LE8 levels. These factors are known to affect health behaviors, cardiometabolic profiles, and CVD risk. Analyses were performed using STATA/SE (17.0; StataCorp LLC) and R (version 4.3.1; R Core Team, R Foundation for Statistical Computing).

Given the sensitivity of CM questions and the likelihood that participants may hesitate to disclose their experiences, *“*prefer not to say*”* responses were treated as “yes” in the sensitivity analysis, and linear regression models were conducted accordingly. To account for the potential co-occurrence of different maltreatment types, additional models, including all specific CM events, were run to assess the robustness of the findings. The standardized LE8 scores were applied to examine the consistency of the association across different LE8 components. The association between cumulative CM events and LE8 levels was further examined using multinomial logistic regression, treating categorical LE8 levels (low, moderate, and high) as the outcome variable.

## Results

### Baseline characteristics of study participants by sex

Among the 153,399 participants, the average age was 55.9 ± 7.7 years), with women being slightly younger than men (55.4 vs 56.5 years. Most of the sample was White (96.8%), showing no significant sex differences (Table 1). More than half of the participants had a college or university degree (50.4%), with a higher proportion of men (54.9%) compared to women (47.0%). Women were more likely to live in areas with a higher TDI score than men. Men were more likely to experience physical abuse, whereas women were more likely to experience other forms of maltreatment, including physical neglect, sexual abuse, emotional abuse, and emotional neglect. In addition, men were more likely to experience only 1 type of CM, whereas women were more likely to experience multiple (≥2) events. However, women had higher LE8 scores with a higher prevalence of moderate-to-high LE8 levels. The prevalence of baseline CVD events and somatic conditions was lower in women than in men.

**Table 1:** Baseline Characteristics of Study Participants by Sex

	Total (N = 153,399)	Men (n = 67,015)	Women (n = 86,384)	*P* Value
Age, y, mean (±SD)	55.9 (±7.7)	56.5 (±7.8)	55.4 (±7.7)	<0.001
Ethnicity, n (%)				0.268
White	148,662 (96.9)	64,906 (96.9)	83,756 (97.0)	
Non-White	4,736 (3.1)	2,108 (3.1)	2,628 (3.0)	
Missing	1 (0.0)	1 (0.0)	0 (0.0)	
Education category, n (%)				<0.001
Low	46,312 (30.2)	18,745 (28.0)	27,567 (31.9)	
Medium	28,355 (18.5)	10,895 (16.2)	17,460 (20.2)	
High (College or University degree, NVQ or HND or HNC or equivalent)	77,377 (50.4)	36,807 (54.9)	40,570 (47.0)	
Missing	1,355 (0.9)	568 (0.9)	787 (0.9)	
Townsend Deprivation Index, n (%)				<0.001
1st quartile (least deprived)	43,258 (28,2)	19,537 (29.2)	23,721 (27.5)	
2nd quartile	40,331 (26.3)	17,716 (26.4)	22,615 (26.2)	
3rd quartile	38,743 (25.3)	16,535 (24.7)	22,208 (25.7)	
4th quartile (most deprived)	30,863 (20.1)	13,133 (19.6)	17,730 (20.5)	
Missing	204 (0.1)	94 (0.1)	110 (0.1)	
Childhood maltreatment events, n (%)				
Physical abuse	28,754 (18.7)	14,057 (21.0)	14,688 (17.0)	<0.001
Physical neglect	24,860 (16.2)	10,143 (15.1)	14,717 (17.0)	<0.001
Sexual abuse	13,427 (8.8)	3,901 (5.8)	9,526 (11.0)	<0.001
Emotional abuse	23,616 (15.4)	8,457 (12.6)	15,159 (17.6)	<0.001
Emotional neglect	33,950 (22.1)	14,332 (21.4)	19,618 (22.7)	<0.001
Number of childhood maltreatment, n (%)				<0.001
None	82,303 (53.6)	36,162 (53.9)	46,141 (53.4)	
1	39,148 (25.5)	18,079 (27.0)	21,069 (24.4)	
2	17,455 (11.4)	7,609 (11.4)	9,846 (11.4)	
≥3	14,493 (9.5)	5,165 (7.7)	9,328 (10.8)	
LE8 score, mean (±SD)	66.2 (±12.3)	63.6 (±11.9)	68.2 (±12.2)	<0.001
Behavioral component score, mean (±SD)	67.8 (±15.2)	66.1 (±15.6)	69.2 (±14.8)	<0.001
Health component score, mean (±SD)	64.6 (±17.5)	61.2 (±16.1)	67.3 (±18.1)	<0.001
LE8 categories, n (%)				<0.001
High (80-100 score)	21,387 (13.9)	5,679 (8.5)	15,708 (18.2)	
Moderate (50-79 score)	117,274 (76.5)	52,971 (79.0)	64,303 (74.4)	
Low (<50)	14,738 (9.6)	8,365 (12.5)	6,373 (7.4)	
Behavioral component categories, n (%)				<0.001
High (80-100 score)	34,881 (23.3)	13,277 (20.2)	21,604 (25.6)	
Moderate (50-79 score)	97,356 (64.8)	43,098 (65.4)	54,258 (64.4)	
Low (<50)	17,927 (11.9)	9,482 (14.4)	8,445 (10.0)	
Health component categories, n (%)				<0.001
High (80-100 score)	29,449 (19.5)	7,946 (12.0)	21,503 (25.5)	
Moderate (50-79 score)	90,169 (59.9)	42,138 (63.7)	48,031 (56.9)	
Low (<50)	30,991 (20.6)	16,086 (24.3)	14,905 (17.6)	
Charlson Comorbidity Index, n (%)				<0.001
None	139,029 (90.6)	60,328 (90.0)	78,701 (91.1)	
Mild	12,511 (8.2)	5,814 (8.7)	6,697 (7.7)	
Moderate	1,084 (0.7)	585 (0.9)	499 (0.6)	
Severe	775 (0.5)	288 (0.4)	487 (0.6)	
CVD before/at baseline, n (%)				
Myocardial infarction	2,225 (1.5)	1,830 (2.7)	395 (0.5)	<0.001
Cerebrovascular disease	1,624 (1.1)	946 (1.4)	678 (0.8)	<0.001
Congestive heart failure	345 (0.2)	273 (0.4)	72 (0.1)	<0.001
Peripheral vascular disease	498 (0.3)	360 (0.5)	138 (0.2)	<0.001

The normality of continuous variables was tested using the Shapiro-Wilk test before applying parametric methods. Baseline characteristics of study participants were compared by sex using a *t*-test for continuous variables and a chi-square test for categorical variables.

CVD = cardiovascular disease; HNC = higher national certificate; HND = higher national diploma; LE8 = Life’s Essential 8; NVQ = national vocational qualification.

### Overall and sex-specific associations between CM and LE8 scores

**Figure 1:**
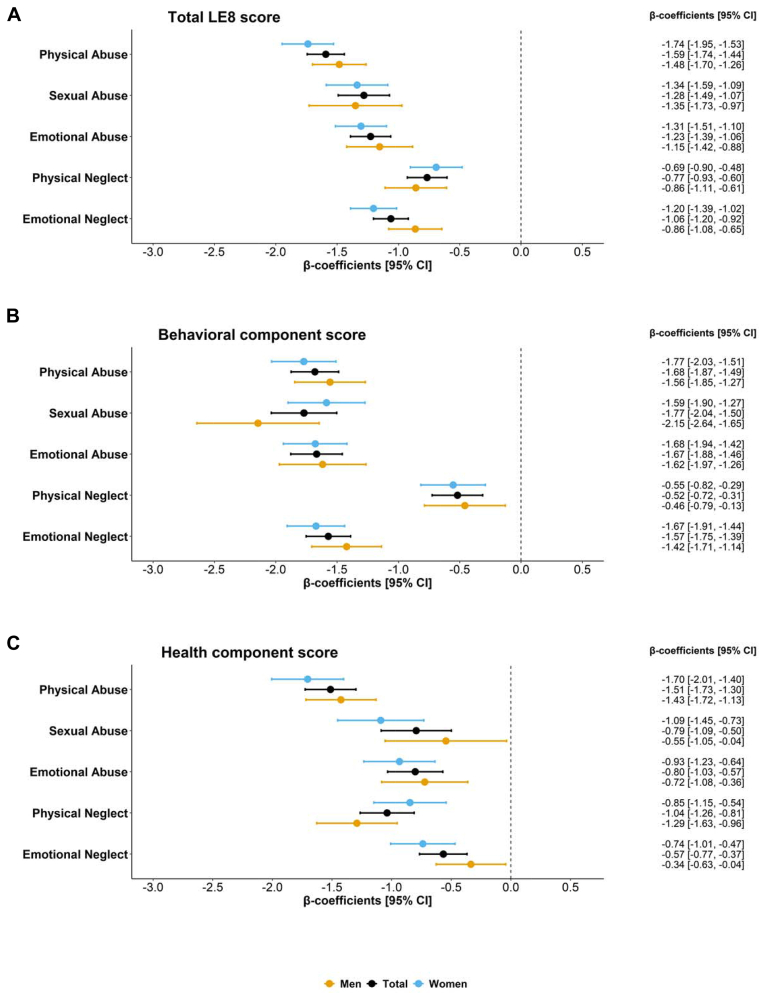
**β-Coefficients of Life’s Essential 8 Scores in Relation to Specific Childhood Maltreatment Events** All models in the total sample were adjusted for age, sex, ethnicity, education, Townsend Deprivation Index, and Charlson Comorbidity Index, whereas sex-specific models adjusted for all except sex. Multivariable linear regression models were applied. LE8 = Life’s Essential 8.

In our total sample analysis, all forms of CM events were significantly associated with lower scores in total-, behavior-, and health-LE8 scores (Figures 1A to 1C). The strongest unfavorable association with the total LE8 score was observed for physical abuse (β-coefficient = −1.59; 95% CI: −1.74 to −1.44), followed by sexual abuse (β = −1.28; 95% CI: −1.49 to −1.07), emotional abuse (β = −1.23; 95% CI: −1.39 to −1.06), emotional neglect (β = −1.06; 95% CI: −1.20 to −0.92), and physical neglect (β = −0.77; 95% CI: −0.93 to −0.60). All forms of maltreatment were significantly associated with both behavioral (Figure 1B) and health (Figure 1C) component scores, but with slightly different patterns. We did not observe any significant interactions between CM events and sex in relation to LE8 scores. When analyses were stratified by sex, similar event-specific associations between CM and LE8 scores were observed as in the total sample. The associations between most types of CM and LE8 scores were slightly stronger in women than in men, although all sex-specific associations were statistically significant.

When examining the relationship between the number of CM events and LE8 scores, we observed that LE8 scores declined sharply with an increasing number of maltreatment events (Table 2). Compared to individuals with no reported CM events, those with 1 event had a significantly lower total LE8 score (β: −0.58; 95% CI: −0.72 to −0.44). The average score decreased further for those with 2 events (β: −1.17; 95% CI: −1.36 to −0.97) and was approximately 4 times lower for those with 3 or more events (β: −2.26; 95% CI: −2.47 to −2.05), suggesting an exponential effect. The health behavior and health factor component scores followed a similar pattern.

**Table 2:** β-Coefficients (95% CI) of Life's Essential 8 Scores in Relation to Cumulative Childhood Maltreatment Numbers

CM events	Total sample	Men	Women
β-Coefficients (95% CI) of Total LE8 Score			
None	Reference	Reference	Reference
1	−0.58 [−0.72 to −0.44]^a^	−0.64 [−0.84 to −0.43]^a^	−0.53 [−0.73 to −0.34]^a^
2	−1.17 [−1.36 to −0.97]^a^	−1.07 [−1.36 to −0.78]^a^	−1.26 [−1.51 to −1.00]^a^
3 or more	−2.26 [−2.47 to −2.05]^a^	−2.40 [−2.74 to −2.06]^a^	−2.23 [−2.50 to −1.96]^a^
*P* for trend	*<0.05*	*<0.05*	*<0.05*
β-Coefficients (95% CI) of Behavioral Component Score			
None	Reference	Reference	Reference
1	−0.84 [−1.02 to −0.66]^a^	−0.88 [−1.16 to −0.61]^a^	−0.80 [−1.04 to −0.56]^a^
2	−1.57 [−1.81 to −1.32]^a^	−1.48 [−1.87 to −1.10]^a^	−1.61 [−1.93 to −1.29]^a^
3 or more	−2.66 [−2.93 to −2.40]^a^	−2.76 [−3.21 to −2.31]^a^	−2.58 [−2.91 to −2.25]^a^
*P* for trend	*<0.05*	*<0.05*	*<0.05*
β-Coefficients (95% CI) of Health Component Score			
None	Reference	Reference	Reference
1	−0.32 [−0.52 to −0.12]^a^	−0.40 [−0.69 to −0.12]^a^	−0.24 [−1.51 to −0.37]^a^
2	−0.77 [−1.04 to −0.50]^a^	−0.68 [−1.07 to −0.19]^a^	−0.90 [−1.27 to −0.54]^a^
3 or more	−1.90 [−2.19 to −1.60]^a^	−2.10 [−2.56 to −1.63]^a^	−1.90 [−2.28 to −1.52]^a^
*P for trend*	*<0.05*	*<0.05*	*<0.05*

Linear regression models for the total sample were adjusted for age, sex, ethnicity, education, Townsend Deprivation Index, and Charlson Comorbidity Index, while sex-specific models adjusted for all except sex. When *P* for trend is significant, it indicates that an inverse dose-response relationship is observed between the number of childhood maltreatment types and Life’s Essential 8 scores.

CM = childhood maltreatment; other abbreviation as in Table 1.

a *P* < 0.01.

Men and women showed similar dose-response patterns in the association between the number of CM events and LE8 scores. Despite men reporting fewer cumulative events with 3 or more occurrences than women, the association between experiencing 3 or more CM events and LE8 scores—particularly the behavioral score—was similar for both men and women (β for men: −2.76, 95% CI: −3.21 to −2.31; for women: −2.58, 95% CI: −2.91 to −2.25). However, the synergistic impact of CM on the health score seems to be more substantial for women, with the association becoming steeper as the number of events increases from 1 to 2 and beyond (β_n = 1_: −0.24, 95% CI: −1.51 to −0.37; β_n = 2_: −0.90, 95% CI: −1.27 to −0.54; β_n = 3+_: −1.90, 95% CI: −2.28 to −1.52).

### Counteracting effect of LE8 levels on the sex-specific association between CM and CVD events

Among the 133,643 participants in the analytical sample for the association between CM and CVD events, 14,907 developed CVD events over an average 13.4-year follow-up period (SD = 1.8 years), including 3,651 cases of MI, 4,587 cases of cerebrovascular disease, 2,768 cases of CHF, and 3,091 cases of peripheral vascular disease (Supplemental Table 5). Compared to individuals with no reported CM, having 3 or more events was associated with an increased risk of MI in women, a higher risk of CHF in men, and elevated risks of cerebrovascular and peripheral vascular disease in both sexes. Detailed information and sex-specific associations between CM and CVD risk are provided in Supplemental Tables 6 and 7. Stratification analysis of CM numbers and all CVD events by total LE8 levels is presented in Supplemental Table 8.

**Figure 2:**
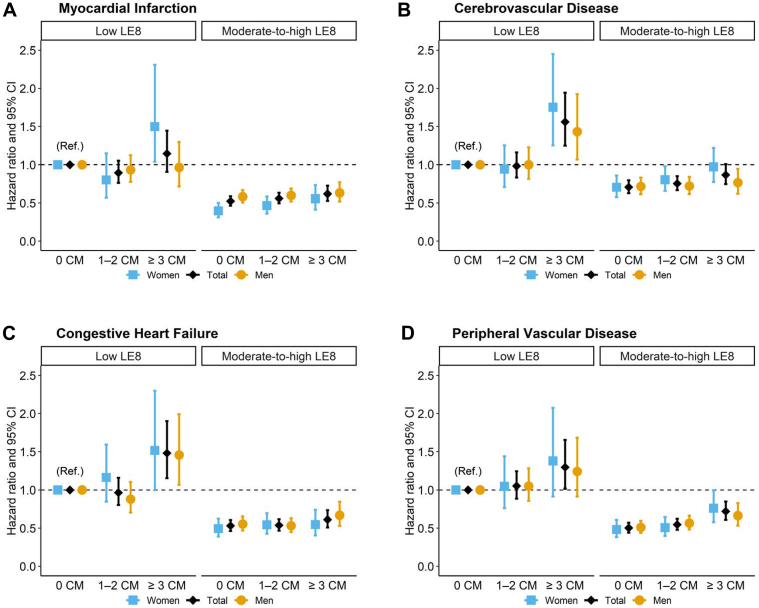
**Childhood Maltreatment Numbers and Incident Cardiovascular Disease by Life’s Essential 8 Levels and Sex** Cox-proportional hazard regression models for the total sample were adjusted for age, sex, ethnicity, education, TDI, and CCI, whereas sex-specific models were adjusted for all except sex. Figure displays the associations for (A) myocardial infarction, (B) cerebrovascular disease, (C) congestive heart failure, and (D) peripheral vascular disease. CM = childhood maltreatment; other abbreviation as in Figure 1.

We further constructed a joint variable that combines the number of CM events (0, 1—2, ≥3) with total LE8 levels (low vs moderate-to-high), resulting in 6 categories: 3 categories for the low LE8 group (0 events, 1—2 events, ≥3 events) and 3 for the moderate-to-high LE8 group (0 events, 1—2 events, ≥3 events). Compared to individuals with no CM and low LE8, the risk of most CVD was significantly higher for those with ≥3 CM events and low LE8 (Figure 2, Central Illustration to 2D). In addition, compared to the group with no CM and low LE8, all 3 groups with moderate-to-high LE8 levels exhibited significantly lower CVD risk, regardless of the number of CM events. Similar patterns were observed for behavior and health components (Supplemental Figures 2 and 3).

Such a modifying effect of moderate-to-high LE8 levels on CM and CVD risk was observed in both men and women. Notably, in those with low LE8 scores, the impact of having 3 or more CM events on MI risk differed by sex. Women showed a significantly increased MI risk when experiencing ≥3 CM events combined with a low LE8 score, whereas this association was not significant in men (Figure 2A). A similar pattern was observed for MI in women with a low behavioral score (Supplemental Figure 2) and for cerebrovascular disease in women with a low health score (Supplemental Figure 3). In contrast, these associations were not significant in men. The proportional hazards assumption was satisfied, and no evidence of collinearity was observed in the models described above.

### Sensitivity analysis

The association between the LE8 scores and CM remained the same after treating participants’ responses “prefer not to say” for CM as “yes” (Supplemental Table 9). When all types of CM events were introduced in a model, the results remained consistent (Supplemental Figure 4). When we standardized LE8 scores for comparison across outcomes, the results were consistent with the main analysis (Supplemental Table 10). Similar results were observed when LE8 scores were treated as categorical outcomes in the multinomial logistic regressions (Supplemental Figures 5 and 6).

## Discussion

The findings of the current study can be summarized as follows: 1) all forms of CM were associated with lower LE8 scores in both men and women, and a higher number of CM events was linked to a greater reduction in LE8 scores; and 2) maintaining a moderate-to-high LE8 level mitigated the harmful effects of CM events on CVD risk for both sexes. However, women who experienced 3 or more CM events and failed to maintain at least a moderate LE8 level had a higher risk of CVD than men, particularly for MI and cerebrovascular diseases.

Although the long-term consequences of adverse early life events on the onset of CVD have been extensively studied, efforts to seek and implement preventive strategies remain insufficient.^23^ In line with our findings, consistent evidence links CM to cardiometabolic outcomes, with substantial research showing associations between childhood adversity and conditions such as hypertension, obesity, type 2 diabetes, and CVD across the life course.^24^^,^^25^ Same evidence exists for behavioral factors, with all forms of CM being linked to unhealthy behaviors (physical inactivity, smoking, unhealthy diet, and inadequate sleep) in adulthood, with the strongest association for physical abuse.^11^^,^^12^^,^^26^^,^^27^ Because all these factors are highly interrelated and often co-occur, it is crucial to evaluate the long-term consequences of CM on aggregated behavioral and health scores.^15^^,^^28^^,^^29^ To the best of our knowledge, this is the first study to assess the long-term consequences of CM on LE8 scores, with findings that are also consistent with other cardiovascular health scores, such as Life’s Simple 7.^15^^,^^28^^,^^29^

Previous research has highlighted the need for sex-specific, adversity-informed approaches to CVD prevention in adulthood, as the modifying effect of sex on the association between CM events and cardiovascular health has been observed but remains insufficiently studied.^3^^,^^15^ In the context of cardiometabolic health, particularly obesity, studies consistently show that women are more strongly affected by CM than men.^16^^,^^25^^,^^30^^,^^31^ However, when both behavioral and cardiometabolic cardiovascular health metrics were examined together, the results were inconclusive. For example, a study from the United States’ II Biomarker Project found that men exhibited a stronger association between childhood adverse events and reduced cardiovascular health compared to women.^15^ Other studies did not observe the supposed sex-specific association but attributed the lack of findings to limitations in statistical power.^30^^,^^32^, ^33^, ^34^ When we separated behavioral and health/cardiometabolic profiles, our findings indicated that men were more influenced by sexual abuse in terms of behavioral scores than women. In contrast, compared to men, women showed a more significant impact from all forms of CM (except for physical neglect) on health/cardiometabolic profile.

Evidence has consistently established a link between childhood adversity, particularly the experience of multiple forms of adversity, and an increased risk of CVD in adulthood,^35^ with elevated CVD risk among women.^36^ Building on previous findings, we further explored the counteracting effect of maintaining moderate-to-high LE8 levels on the relationship between CM and specific CVD outcomes. Interestingly, we observed that individuals who maintained moderate-to-high levels of LE8 despite experiencing CM showed a substantial reduction in all CVD events, compared to those who reported no CM but had low LE8 levels. This counteracting effect appeared to benefit both men and women. Among individuals with low LE8 scores, the highest CVD risk was observed in those who had experienced 3 or more types of CM, with the effect being especially pronounced in women. Similar associations were found when the LE8 score was analyzed across its behavioral and health components.

Using the same U.K. Biobank data set, previous studies have demonstrated that the pathways linking CM events to CVD risk involve mental health status, behaviors, metabolic factors (BMI and cholesterol levels), and inflammation.^18^^,^^37^ CM may increase vulnerability to anxiety and depression by impairing emotional regulation and cognitive capacity, which in turn can lead to maladaptive coping strategies, including risky behaviors and social isolation.^38^ Furthermore, the link between CM and lower LE8 metabolic score may involve dysregulation of the hypothalamic-pituitary-adrenal (HPA) axis, which controls cortisol, an anti-inflammatory glucocorticoid hormone, essential in maintaining cardiometabolic stability by adapting to stressors.^39^ The sex differences in CVD outcomes in response to CM may be partially attributed to biological differences between men and women. The decline in estrogen levels after menopause may contribute to an increased vulnerability to CVD in women.^40^ Furthermore, cardiovascular homeostasis and the body’s stress response mechanisms exhibit sex-based differences, with distinct stress-coping strategies for men and women via the HPA axis.^41^ These sex-specific differences in the HPA stress system may partly underlie disparities in CVD development.^41^ These theoretical frameworks may help explain the observed sex differences in cardiometabolic profiles, health-related behaviors, and CVD risk in response to CM.

### Strengths and limitations

This study possesses several notable strengths. Firstly, it uses the extensive U.K. Biobank database, with a longitudinal design, which offers comprehensive lifestyle, health, and sociodemographic data, ensuring a large sample size and robust statistical power. Cardiometabolic measures were objectively assessed, enhancing the validity of the findings. Secondly, the LE8 score provides an additional sleep assessment than the previous AHA’s recommendation.^15^ Thirdly, the CTS is a validated instrument that provides a reliable assessment of CM. However, the U.K. Biobank is a volunteer-based prospective cohort study, so caution is warranted when interpreting the generalizability of our findings. CM data collected retrospectively via online questionnaires were administered after baseline data collection. Potential selection and reporting bias may be introduced, as only one-third of the cohort completed the CM questionnaire. The participants in our analytical sample were younger, had higher LE8 scores, and had lower comorbidity than those excluded (Supplemental Table 11). In addition, exposure to CM is linked to poor mental health and mortality.^38^ This may contribute to the dilution of effect estimates in the association between CM and LE8. Furthermore, this study may introduce nondifferential exposure misclassification due to recall bias, which may lead to an underestimation of effect estimates.^42^ The CCI measurements may underestimate the actual burden of comorbidity because of their limited sensitivity and health conditions. Considering that CM is one of the social determinants of health and may be influenced by factors such as economic instability, lower parental educational attainment, and neighborhood deprivation, future research should include a broader range of early-life adversities to achieve a more comprehensive understanding.

## Conclusions

In the current study, we found that experiencing CM was associated with unfavorable LE8 scores across both behavioral and health components in adult men and women, and this association became stronger with an increasing number of CM events. Nevertheless, maintaining a moderate-to-high LE8 score in adulthood substantially reduced CVD risk, regardless of CM exposure. Women, compared to men, were more vulnerable to MI and cerebrovascular diseases in the presence of cumulative CM exposure combined with a low LE8 score. This study explains, at least partly, both how CM leads to adverse CVD outcomes and why women may experience worse outcomes. Our findings additionally convey a positive message: the impact of such early-life adversities can be offset by adopting good lifelong lifestyle practices. It could help services identify individuals, particularly women, who have experienced CM and implement interventions to improve their opportunity, capability, and motivation to enhance LE8.

Perspectives **COMPETENCY IN MEDICAL KNOWLEDGE AND COMMUNICATION SKILLS:** This study underscores the protective role of adhering to LE8 recommendations in mitigating CVD risk associated with CM. Our findings indicate that women are more vulnerable than men to CVD risk when exposed to both CM and poor LE8 adherence. These results highlight the importance of patient-centered, trauma-informed care that addresses both behavioral and psychosocial factors in cardiovascular risk management.

**TRANSLATIONAL OUTLOOK:** Currently, integrating trauma-informed health care practices into CVD prevention remains underexplored. Promoting LE8 adherence helps prevent CVD, even among individuals with a history of CM. This is particularly important for women, as it offers substantial protection against the harmful cardiovascular consequences of early-life adversity compared with those who do not follow the guidelines. Future research should explore strategies to promote LE8 adherence, particularly among women with a history of childhood adversity, by enhancing their opportunities, capabilities, and motivation to maintain optimal cardiovascular health.

## Funding support and author disclosures

Dr R. Wang has received research funding from the 10.13039/501100004359Swedish Research Council (VR: 2016-06658, 2022-01404), the 10.13039/501100003170Knowledge Foundation (KK-stiftelsen: 2022-0202, 20240066), Foundation for Geriatric Diseases at 10.13039/501100004047Karolinska Institutet (2022-01286) and Demensförbundet 2024. Dr H.-X. Wang has received funding from VR (2023-02601) and the 10.13039/501100006636Swedish Research Council for Health, Working Life, and Welfare (FORTE: 2019-01120, 2020-00313). All other authors have reported that they have no relationships relevant to the contents of this paper to disclose.
