# Supplementary material for: Childhood Maltreatment and Cardiovascular Health: Role of Sex and Life’s Essential 8 in the U.K. Biobank
Source: JACC Adv. 2026 Jan 28;5(1):102483. doi: 10.1016/j.jacadv.2025.102483 (PMC12869877; doi:10.1016/j.jacadv.2025.102483)
Supplement: Supplemental Material [file mmc1.docx]

**Supplemental Appendix**

Supplement for “Childhood Maltreatment and Cardiovascular Health: Role of Sex and Life’s Essential 8 in the UK-Biobank” by Bucha and Li et al.

**Table of Contents**

[Supplemental Table 1: Questions and response variables for assessing childhood maltreatment in the UK Biobank 3](#_Toc212818179)

[Supplemental Table 2: Defining and quantifying metrics of Life's Essential 8, Cardiovascular Health 4](#_Toc212818180)

[Supplemental Table 3: Criterion of a Healthy Diet in the UK Biobank 6](#_Toc212818181)

[Supplemental Table 4: ICD-10 codes used for assessing the Charlson Comorbidity Index 8](#_Toc212818182)

[Supplemental Table 5: Incident CVD cases and follow-up 9](#_Toc212818183)

[Supplemental Table 6: Association between childhood maltreatment events and CVD events 10](#_Toc212818184)

[Supplemental Table 7: Association between Childhood maltreatment numbers and CVD events 11](#_Toc212818185)

[Supplemental Table 8: Association between cumulative childhood maltreatment number and CVD events by LE8 category 12](#_Toc212818186)

[Supplemental Table 9: Association between childhood maltreatment numbers and LE8 and its components 13](#_Toc212818187)

[Supplemental Table 10: Association between childhood maltreatment events and standardized z-scores of LE8 and its components 14](#_Toc212818188)

[Supplemental Table 11: Socio-demographic and health-related characteristics of participants included and excluded in the study 15](#_Toc212818189)

[Supplemental Table 12: Association between LE8 scores and CVD risk 16](#_Toc212818190)

[Supplemental Table 13: Association between LE8 score and All-Cause Mortality 17](#_Toc212818191)

[Supplemental Figure 1: Flow-chart of study participants in the UK Biobank 18](#_Toc212818192)

[Supplemental Figure 2: Association between childhood maltreatment and incident CVD events by behavioral component levels and sex 19](#_Toc212818193)

[Supplemental Figure 3: Association between childhood maltreatment and incident CVD events by health component and sex 20](#_Toc212818194)

[Supplemental Figure 4: β-coefficients of LE8 scores in relation to specific childhood maltreatment events 21](#_Toc212818195)

[Supplemental Figure 5: Association between cumulative childhood maltreatment numbers and categories of LE8 22](#_Toc212818196)

[Supplemental Figure 6: Association between cumulative childhood maltreatment numbers and LE8 categories by sex 23](#_Toc212818197)

[References 24](#_Toc212818198)

## Supplemental Table 1: Questions and response variables for assessing childhood maltreatment in the UK Biobank

| **Childhood maltreatment** | **Field ID** | **Questions** | **Response/Description** | **Cut off** |
| --- | --- | --- | --- | --- |
| Physical abuse | 29077 | Physically abused by family as a child. | 0, Never true; 1, Rarely true; 2, Sometimes true; 3, Often; 4, Very often true | ≥1 |
| Physical neglect | 29080 | Someone to take them to doctor when needed as a child. | 0, Never true; 1, Rarely true; 2, Sometimes true; 3, Often; 4, Very often true | ≤3 |
| Sexual Abuse | 29079 | Sexually molested as a child. | 0, Never true; 1, Rarely true; 2, Sometimes true; 3, Often; 4, Very often true | ≥1 |
| Emotional Abuse | 29078 | Felt hated by family member as a child. | 0, Never true; 1, Rarely true; 2, Sometimes true; 3, Often; 4, Very often true | ≥1 |
| Emotional Neglect | 29076 | Felt loved as a child. | 0, Never true; 1, Rarely true; 2, Sometimes true; 3, Often; 4, Very often true | ≤2 |
| Cumulative childhood maltreatment |  | Summary score of childhood maltreatment | Summary score of five childhood maltreatment items (0-5) | - |

## Supplemental Table 2: Defining and quantifying metrics of Life's Essential 8, Cardiovascular Health

| **Metrics** | **UK Biobank Field** | **Scores** | **Definitions** |
| --- | --- | --- | --- |
| **Behavioral component metrics** | |  |  |
| Diet* | Category 100052, mentioned in the Appendix Table 3 | 100 | Diet score ≥95th percentile |
|  |  | 80 | 75th – <95th percentiles |
|  |  | 50 | 50th – <75th percentiles |
|  |  | 25 | 25th – <50th percentiles |
|  |  | 0 | 1st – <25th percentiles |
| Physical activity | 22038 and 22039 | 100 | Metabolic equivalent task minutes per week for moderate/vigorous activity ≥150 |
|  |  | 90 | 120 – <150 |
|  |  | 80 | 90 – <120 |
|  |  | 60 | 60 – <90 |
|  |  | 40 | 30 – <60 |
|  |  | 20 | 1 – <30 |
|  |  | 0 | 0 |
| Nicotine exposure* | 20116, 22507, 2897, 6194, 20003, 1269 | 100 | Never smoker |
|  |  | 75 | Former smoker, quit > 5 years, or missing quitting years |
|  |  | 50 | Former smoker, quit 1–5 years |
|  |  | 25 | Former smoker, quit <1 year, or currently using nedocromil sodium |
|  |  | 0 | Current smoker |
|  |  | Subtract 20 points (unless the score is 0) for living with an active indoor smoker at home | |
| Sleep health* | 1160 | 100 | Sleep duration each 24 hours: 7- <9 hours |
|  |  | 90 | 9 – <10 hours |
|  |  | 70 | 6 – <7 hours |
|  |  | 40 | 5 – <6 or ≥10 hours |
|  |  | 20 | 4 – <5 hours |
|  |  | 0 | <4 hours |
| **Health component metrics** | |  |  |
| Body mass index | 21001 | 100 | <25 kg/m2 |
|  |  | 70 | 25 – <30 kg/m2 |
|  |  | 30 | 30 – <35 kg/m2 |
|  |  | 15 | 35 – <40 kg/m2 |
|  |  | 0 | ≥40 kg/m2 |
| Blood lipids | 30690, 30760, 6177 | 100 | Non-HDL-cholesterol <130 mg/dL |
|  |  | 60 | 130 – <160 mg/dL |
|  |  | 40 | 160 – <190 mg/dL |
|  |  | 20 | 190 – <220 mg/dL |
|  |  | 0 | ≥220 mg/dL |
|  |  | Subtract 20 points for drug-treating level | |
| Blood glucose* | 2443, 30740, 30750 | 100 | No history of diabetes and blood glucose <100 mg/dL (or HbA1c < 39 mmol/mol) |
|  |  | 60 | No diabetes and blood glucose 100 – 125 mg/dL (or HbA1c 39 – 46 mmol/mol) |
|  |  | 40 | Diabetes with HbA1c < 53 mmol/mol |
|  |  | 30 | Diabetes with HbA1c 53 – <64 mmol/mol |
|  |  | 20 | Diabetes with HbA1c 64 – <75 mmol/mol |
|  |  | 10 | Diabetes with HbA1c 75 – <86 mmol/mol |
|  |  | 0 | Diabetes with HbA1c ≥86 mmol/mol |
| Blood pressure | 4080, 93, 4079, 94, 6177 | 100 | SBP <120 mmHg, and DBP < 80 mmHg |
|  |  | 75 | SBP 120 – <130 mmHg, and DBP < 80 mmHg |
|  |  | 50 | SBP 130 – <140 mmHg, or DBP 80 – <90 mmHg |
|  |  | 25 | SBP 140 – <160 mmHg, or DBP 90 – <100 mmHg |
|  |  | 0 | SBP ≥160 mmHg, or DBP ≥ 100 mmHg |
|  |  | Subtract 20 points for drug-treating level. | |

Abbreviations: HDL= high-density lipoprotein, HbA1C=glycated hemoglobin, SBP=systolic blood pressure,DBP=diastolic blood pressure.

*Modified from the recommendation of the American Heart Association in 2022.1

Modified items: (1) When defining diet habits, we used the diet points assessed from 12 food items, according to Beydoun’s study,2 but not the Healthy Eating Index-2015 or the Index of Dietary Approaches to Stop Hypertension which were recommended by the American Heart Association. (2) When defining nicotine exposure, people who quit smoking while missing the information regarding quitting years were assigned a score of 50. (3) When defining sleep health, our study used the total sleep duration of 24 hours, which is different from the average hours of sleep per night recommended by the American Heart Association. (4) When defining blood glucose, our study used the level of HbA1c with a unit of mmol/mol, but not %.

*Notes.* To avoid discarding partially complete data, the proration method was applied to calculate the behavioral component, health component, and total LE8 scores.2 Specifically, we assigned participants with missing more than half of the metrics within a component a missing value. If participants with missing less than half of the metrics, a prorated score was calculated. A similar proration method was used for diet scores, with detailed scoring provided in Supplemental Table 3.

## Supplemental Table 3: Criterion of a Healthy Diet in the UK Biobank

| **Food item** | **UK Biobank Field** | **Definition of meeting criterion** | **Criteria scoring** | **Interpretation of serving size** |
| --- | --- | --- | --- | --- |
| **Consume more** |  |  |  |  |
| Fresh and dried fruits | 1309 and 1319 | Consumption of fresh and dried fruits ≥3 servings per day. | 1= meets criteria, 0= does not meet criteria | One serving of fresh fruits = one piece. One serving of dried fruits = 3 pieces. |
| Salad/raw and cooked vegetables | 1289 and 1299 | Consumption of salad/raw and cooked vegetables ≥3 servings per day. | 1= meets criteria, 0= does not meet criteria | One serving of cooked vegetables = 8 tablespoons. One serving of salad/raw vegetables = 12 tablespoons. |
| Whole grains |  | ≥3 servings per day of bread or cereal | 1= meets criteria, 0= does not meet criteria |  |
| Bread | 1438 and 1448 | Daily slices of wholemeal or wholegrain bread |  | One serving equals 1 slice of bread |
| Cereal* | 1458 and 1468 | Daily bowls of whole wheat cereal (bran cereal, biscuit cereal, oat cereal, and muesli) |  | One serving equals 1 bowl of cereal |
| Fish shellfish: Oily and non-oily fish | 1329 and 1339 | Consumption of oily and non-oily fish shellfish ≥2 servings/week | 1= meets criteria, 0= does not meet criteria |  |
| Dairy products (cheese)* | 1408 | Reporting consumption of cheese once a day. | 1= meets criteria, 0= does not meet criteria |  |
| Vegetable oil | 2654 | Reporting use of olive oil or polyunsaturated/sunflower oil | 1= meets criteria, 0= does not meet criteria |  |
| **Consume less** |  |  |  |  |
| Refined grains, starches, added sugars | 1438 and 1448 | Non-whole grains <1.5 servings per day | 1= meets criteria, 0= does not meet criteria |  |
| Processed meats | 1349 | Once a week or less. | 1= meets criteria, 0= does not meet criteria |  |
| Unprocessed red meats | 1369, 1379, and 1389 | Summation of frequency of consumption across three types of red meats (lamb/mutton, beef or pork) <3. | 1= meets criteria, 0= does not meet criteria |  |
| Industrial trans fat | 1428 | Never or rarely using spread. | 1= meets criteria, 0= does not meet criteria |  |
| Sugar-sweetened beverages | 6144 | Never eating sugar or food/drink containing sugar. | 1= meets criteria, 0= does not meet criteria |  |
| Sodium | 1478 | Never or rarely adding salad to food. | 1= meets criteria, 0= does not meet criteria |  |
| **Total score** | | **Baseline sample to define dietary score** | | |
| Range | | 0─12 | | |
| 25th percentile | | 2.18 | | |
| 50^th^ percentile | | 3.6 | | |
| 75^th^ percentile | | 5 | | |
| 95^th^ percentile | | 6.67 | | |

*Modified items: (1) Cereal was defined by variables 1458 and 1468 in our study, while 1458 and 1448 in Beydoun’s study.^2^ (2) The dairy product met the criterion when participants reported the consumption of two milk items and eating cheese once a day, in Beydoun’s study.^2^ However, the number of milk items was not available in UK Biobank. Therefore, we only used the cheese item to define the dairy product.

## Supplemental Table 4: ICD-10 codes used for assessing the Charlson Comorbidity Index

| **Charlson Comorbidity Index (CCI)** | **International Classification of Diseases-10 (ICD-10)** | **Weights** |
| --- | --- | --- |
| Myocardial infarction | I21, I22, I252 | 1 |
| Congestive heart failure | I110, I130, I132, I50 | 1 |
| Peripheral vascular disease | I70, I71, I731, I738, I739, I771, I790, I792, K551, K558, K559, R02, Z958, Z959 | 1 |
| Cerebrovascular disease | I60-I69, G45, G46 | 1 |
| Chronic pulmonary disease | J40-J47, J60, J61, J62, J63, J64, J65, J66, J67, J684, J70, J841, J920, J961, J982 | 1 |
| Connective tissue disease | M05, M06, M30, M315, M32, M33, M34, M351, M353, M360 | 1 |
| Ulcer disease | K25, K26, K27, K28 | 1 |
| Mild liver disease | B18, K700, K701, K702, K703, K709, K713, K714, K715, K717, K73, K74, K760, K762, K763, K764, K768, K769, | 1 |
| Diabetes mellitus | E100, E101, E106, E108, E109, E110, E111, E116, E118, E119, E120, E121, E126, E128, E129, E130, E131, E136, E138, E139, E140, E141, E146, E148, E149, | 1 |
| Hemiplegia | G041, G114, G801, G802, G81, G82, G839, G830, G831, G832, G833, G834 | 2 |
| Moderate/severe renal disease | I120, I131, N032, N033, N034, N035, N036, N037, N052, N053, N054, N055, N056, N057, N18, N19, N250, Z940, Z992 | 2 |
| Diabetes mellitus with chronic complications | E102, E103, E104, E105, E107, E112-E115, E122-E125, E132- E135, E142- E145, E117, E127, E137, E147 | 2 |
| Any tumor | C00-C14, C15-C26, C30-C34, C37-C41, C43, C45-C49, C50-C50, C51- C58, C60-C63, C64-C68, C69-C72, C73-C76, C97 | 2 |
| Leukemia | C91, C92, C93, C94, C95 | 2 |
| Lymphoma | C81, C82, C83, C84, C85, C88, C90, C96 | 2 |
| Moderate/severe liver disease | 85, K704, K72, K766 | 3 |
| Metastatic solid tumor | C77, C78, C79, C80 | 6 |
| AIDS | B20, B21, B22, B23, B24 | 6 |
| Dementia* | Algorithmically defined cases (Fields ID 42018–42025), the first-occurrence data reporting dementia onsets within mental and behavioral disorders (Fields ID 130836–130843), hospital inpatient record (Fields ID 41270-71, 41280-81), death register (Fields ID 40001-02), and primary care data recorded (Field 42040) | 1 |
| Cutoffs for category | None: 0, Mild: 1—2, moderate: 3—4, and severe: ≥5 |  |

*Dementia cases obtained in reference to the study of Hu et al.^3^

Abbreviations: AIDS= acquired immune deficiency syndrome, CCI=charlson comorbidity cndex, ICD= international classification of diseases

## Supplemental Table 5: Incident CVD cases and follow-up

| CVD events | Total | Men | Women | p-value | Average follow-up |
| --- | --- | --- | --- | --- | --- |
| Myocardial Infarction | 3,651 (2.7) | 2,568 (4.5) | 1,083 (1.4) | <0.05 | 13.4 [SD=1.8] |
| Cerebrovascular disease | 4,587 (3.4) | 2,470 (4.3) | 2,117 (2.8) | <0.05 | 13.4 [SD=1.7] |
| Congestive Heart Failure | 2,768 (2.1) | 1,702 (3.0) | 1,066 (1.4) | <0.05 | 13.5 [SD=1.8] |
| Peripheral vascular disease | 3,091 (2.3) | 1,941 (3.4) | 1,150 (1.5) | <0.05 | 13.4 [SD=1.8] |
| Total | 14,097 | 8,681 | 5,416 |  | 13.4 [SD=1.8] |

Abbreviations: SD= standard deviation.

## Supplemental Table 6: Association between childhood maltreatment events and CVD events

| CM event | Hazard Ratio (95% CI) for myocardial infarction | | |
| --- | --- | --- | --- |
|  | Total | Men | Women |
| Physical Abuse | 1.07 [0.98 - 1.16] | 1.01 [0.92 - 1.12] | 1.23 [1.05 - 1.44] ^**^ |
| Physical Neglect | 1.13 [1.04 - 1.22] ^**^ | 1.07 [0.96 - 1.18] | 1.26 [1.09 - 1.45] ^**^ |
| Sexual Abuse | 1.18 [1.05 - 1.33] ^**^ | 1.13 [0.97 - 1.32] | 1.28 [1.07 - 1.53] ^**^ |
| Emotional Abuse | 1.20 [1.10 - 1.32] ^**^ | 1.13 [1.01 - 1.27] ^*^ | 1.35 [1.16 - 1.56] ^**^ |
| Emotional Neglect | 1.13 [1.04 - 1.22] ^**^ | 1.06 [0.97 - 1.17] | 1.28 [1.12 - 1.47] ^**^ |
|  | Hazard Ratio (95% CI) for cerebrovascular disease | | |
|  | Total | Men | Women |
| Physical Abuse | 1.03 [0.95-1.11] | 0.96 [0.87-1.06] | 1.13 [1.01-1.28] ^*^ |
| Physical Neglect | 1.24 [1.15-1.33] ^**^ | 1.20 [1.08-1.33] ^**^ | 1.28 [1.1-1.41] ^**^ |
| Sexual Abuse | 1.18 [1.07-1.31] ^**^ | 1.14 [0.98-1.33] | 1.22 [1.08-1.40] ^**^ |
| Emotional Abuse | 1.25 [1.16-1.36] ^**^ | 1.16 [1.03-1.31] ^**^ | 1.34 [1.20-1.49] ^**^ |
| Emotional Neglect | 1.05 [0.98-1.13] | 1.00 [0.91-1.11] | 1.11 [1.00-1.23] ^*^ |
|  | Hazard Ratio (95% CI) for congestive heart failure | | |
|  | Total | Men | Women |
| Physical Abuse | 1.10 [1.00-1.21] | 1.07 [0.95-1.21] | 1.15 [0.98-1.36] |
| Physical Neglect | 1.15 [1.05-1.26] ^*^ | 1.16 [1.03-1.31] ^*^ | 1.13 [0.98-1.30] |
| Sexual Abuse | 1.13 [0.99-1.29] | 1.18 [0.98-1.42] | 1.09 [0.90-1.32] |
| Emotional Abuse | 1.11 [1.00-1.23] | 1.08 [0.93-1.26] | 1.14 [0.97-1.34] |
| Emotional Neglect | 1.10 [1.01-1.20] ^*^ | 1.06 [0.95-1.20] | 1.15 [1.00-1.32] |
|  | Hazard Ratio (95% CI) for peripheral vascular disease | | |
|  | Total | Men | Women |
| Physical Abuse | 1.18 [1.08-1.29] ^**^ | 1.15 [1.03-1.28] ^*^ | 1.26 [1.09-1.47] ^**^ |
| Physical Neglect | 1.18 [1.08-1.29] ^**^ | 1.15 [1.03-1.28] ^*^ | 1.24 [1.08-1.42] ^**^ |
| Sexual Abuse | 1.22 [1.08-1.38] ^**^ | 1.17 [0.99-1.40] | 1.26 [1.06-1.49] ^**^ |
| Emotional Abuse | 1.34 [1.21-1.47] ^**^ | 1.23 [1.08-1.41] ^**^ | 1.47 [1.28-1.69] ^**^ |
| Emotional Neglect | 1.18 [1.09-1.28] ^**^ | 1.11 [0.99-1.23] | 1.30 [1.14-1.48] ^**^ |

^*^p-value <0.05.

^**^p-value <0.01.

Note: Cox proportional hazard regression models for the total sample were adjusted for age, sex, ethnicity, education qualification, Townsend deprivation index, and Charlson Comorbidity Index, while sex-specific models were adjusted for all except sex.

Abbreviations: CI=confidence interval, CM=childhood maltreatment.

## Supplemental Table 7: Association between Childhood maltreatment numbers and CVD events

|  | Hazard Ratio (95% CI) for myocardial infarction | | |
| --- | --- | --- | --- |
| CM events | Total | Men | Women |
| One | 1.00 [0.92-1.08] | 0.97 [0.89-1.07] | 1.05 [0.91-1.22] |
| Two | 1.16 [1.05-1.29] ^**^ | 1.12 [0.99-1.27] | 1.28 [1.07-1.55] ^**^ |
| Three or more | 1.21 [1.08-1.36] ^**^ | 1.08 [0.94-1.25] | 1.48 [1.24-1.78] ^**^ |
|  | Hazard Ratio (95% CI) for cerebrovascular disease | | |
|  | Total | Men | Women |
| One | 1.03 [0.96-1.11] | 0.98 [0.89-1.07] | 1.10 [0.99-1.22] |
| Two | 1.12 [1.02-1.23] ^*^ | 1.09 [0.96-1.24] | 1.17 [1.02-1.34] ^*^ |
| Three or more | 1.31 [1.18-1.44] ^**^ | 1.17 [1.01-1.35] ^*^ | 1.46 [1.27-1.66] ^**^ |
|  | Hazard Ratio (95% CI) for congestive heart failure | | |
|  | Total | Men | Women |
| One | 0.98 [0.90-1.08] | 0.92 [0.82-1.03] | 1.10 [0.95-1.27] |
| Two | 1.08 [0.96-1.22] | 1.03 [0.88-1.20] | 1.17 [0.97-1.42] |
| Three or more | 1.26 [1.11-1.43] ^**^ | 1.31 [1.11-1.54] ^**^ | 1.21 [0.99-1.48] |
|  | Hazard Ratio (95% CI) for peripheral vascular disease | | |
|  | Total | Men | Women |
| One | 1.07 [0.98-1.17] | 1.10 [0.99-1.23] | 1.01 [0.87-1.16] |
| Two | 1.15 [1.02-1.28] ^*^ | 1.11 [0.96-1.28] | 1.21 [1.00-1.45] |
| Three or more | 1.45 [1.29-1.62] ^**^ | 1.33 [1.14-1.56] ^**^ | 1.58 [1.33-1.88] ^**^ |

^*^ p-value <0.05.

^**^ p-value <0.01.

Note: Cox proportional hazard regression models for the total sample were adjusted for age, sex, ethnicity, education qualification, Townsend deprivation index, and Charlson Comorbidity Index, while sex-specific models were adjusted for all except sex.

Abbreviations: CI=confidence interval, CM=childhood maltreatment.

## Supplemental Table 8: Association between cumulative childhood maltreatment number and CVD events by LE8 category

|  | Hazard Ratio (95% CI) for myocardial Infarction | | |
| --- | --- | --- | --- |
| CM events | Total | Low LE8 | Moderate-to-high LE8 |
| None | Reference | Reference | Reference |
| One | 0.99 [0.91-1.07] | 0.81 [0.67-0.98] | 1.03 [0.95-1.13] |
| Two | 1.14 [1.03-1.27] ^**^ | 1.08 [0.87-1.35] | 1.16 [1.03-1.30]^*^ |
| Three or more | 1.18 [1.05-1.32] ^**^ | 1.10 [0.87-1.39] | 1.19 [1.05-1.36]^**^ |
|  | Hazard Ratio (95% CI) for cerebrovascular disease | | |
|  | Total | Low LE8 | Moderate-to-high LE8 |
| None | Reference | Reference | Reference |
| One | 1.03 [0.96-1.10] | 0.96 [0.79-1.16] | 1.04 [0.96-1.12] |
| Two | 1.11 [1.01-1.22] ^*^ | 1.03 [0.81-1.30] | 1.13 [1.02-1.25]^*^ |
| Three or more | 1.29 [1.17-1.42] ^**^ | 1.55 [1.24-1.194]^**^ | 1.23 [1.10-1.37]^**^ |
|  | Hazard Ratio (95% CI) for congestive heart failure | | |
|  | Total | Low LE8 | Moderate-to-high LE8 |
| None | Reference | Reference | Reference |
| One | 0.98 [0.89-1.07] | 0.91 [0.74-1.13] | 1.00 [0.90-1.10] |
| Two | 1.06 [0.94-1.20] | 1.08 [0.83-1.40] | 1.06 [0.92-1.21] |
| Three or more | 1.23 [1.08-1.40] ^**^ | 1.43 [1.11-1.83]^**^ | 1.16 [1.00-1.35]^*^ |
|  | Hazard Ratio (95% CI) for peripheral vascular disease | | |
|  | Total | Low LE8 | Moderate-to-high LE8 |
| None | Reference | Reference | Reference |
| One | 1.06 [0.98-1.16] | 1.02 [0.84-1.23] | 1.07 [0.98-1.18] |
| Two | 1.12 [1.00-1.25] | 1.14 [0.90-1.45] | 1.11 [0.98-1.27] |
| Three or more | 1.40 [1.25-1.57] ^**^ | 1.32 [1.03-1.69]^*^ | 1.43 [1.25-1.63]^**^ |

^*^p-value <0.05.

^**^ p-value <0.01.

Note: Cox proportional hazard regression models were adjusted for age, sex, ethnicity, education qualification, Townsend deprivation index, and Charlson Comorbidity Index, and further by LE8 category in the total sample.

Abbreviations: CI=confidence interval, CM=childhood maltreatment, LE8=Life’s Essential 8.

## Supplemental Table 9: Association between childhood maltreatment numbers and LE8 and its components

|  | β-coefficients (95% CI) of total LE8 score | | |
| --- | --- | --- | --- |
| CM events | Total sample | Men | Women |
| None | Reference | Reference | Reference |
| One | -0.56 [-0.71, -0.40] ^**^ | -0.73 [-0.93, -0.53] ^*^ | -0.58 [-0.76, -0.39] ^*^ |
| Two | -1.19 [-1.39, -0.99] ^**^ | -1.21 [-1.49, -0.93] ^*^ | -1.38 [-1.62, -1.13] ^*^ |
| Three or more | -2.27 [-2.49, -2.01] ^**^ | -2.55 [-2.88, -2.22] ^*^ | -2.43 [-2.68, -2.18] ^*^ |
| p for trend | *<0.05* | *<0.05* | *<0.05* |
|  | β-coefficients (95% CI) of behavioral component score | | |
| CM events | Total sample | Men | Women |
| None | Reference | Reference | Reference |
| One | -0.83 [-1.02, -0.64] ^**^ | -0.85 [-1.15, -0.56] ^**^ | -0.82 [-1.07, -0.56] ^**^ |
| Two | -1.64 [-1.89, -1.39] ^**^ | -1.55 [-1.96, -1.15] ^**^ | -1.69 [-2.02, -1.36] ^**^ |
| Three or more | -2.65 [-2.92, -2.38] ^**^ | -2.74 [-3.21, -2.27] ^**^ | -2.59 [-2.92, -2.52] ^**^ |
| p for trend | *<0.05* | *<0.05* | *<0.05* |
|  | β-coefficients (95% CI) of health component score | | |
| CM events | Total sample | Men | Women |
| None | Reference | Reference | Reference |
| One | -0.26 [-0.47, -0.53] ^*^ | -0.35 [-0.65, -0.04] ^*^ | -0.19 [-0.48, 0.1] |
| Two | -0.75 [-1.03, -0.47] ^**^ | -0.62 [-1.03, -0.20] ^**^ | -0.89 [-1.27, -0.51] ^**^ |
| Three or more | -1.91 [-2.22, -1.62] ^**^ | -1.94 [-2.42, -146] ^**^ | -1.99 [-2.37, -1.60] ^**^ |
| *P for trend* | *<0.05* | *<0.05* | *<0.05* |
| ^*^p-value <0.05.  ^**^ p-value <0.01.  Note: This is a sensitivity analysis where response to childhood maltreatment questions “prefer not to say” was treated as “Yes”.  All the models were adjusted for age, sex, ethnicity, education qualification, Townsend deprivation index, and Charlson Comorbidity Index.  Abbreviations: CI=confidence interval, CM=childhood maltreatment, LE8=Life’s Essential 8. | | | |

## Supplemental Table 10: Association between childhood maltreatment events and standardized z-scores of LE8 and its components

|  | β-coefficients (95% CI) for total z-standardized LE8 score | | |
| --- | --- | --- | --- |
| CM events | Total sample | Men | Women |
| Physical Abuse | -0.13 [-0.14, -0.12] ^**^ | -0.12 [-0.14, -0.10] ^**^ | -0.14 [-0.16, -0.12] ^**^ |
| Sexual Abuse | -0.10 [-0.12, -0.09] ^**^ | -0.11 [-0.14, -0.08] ^**^ | -0.12 [-0.13, -0.09] ^**^ |
| Emotional Abuse | -0.10 [-0.11, -0.09] ^**^ | -0.09 [-0.12, -0.07] ^**^ | -1.11 [-0.12, -0.09] ^**^ |
| Physical Neglect | -0.06 [-0.76, -0.05] ^**^ | -0.07 [-0.09, -0.05] ^**^ | -0.06 [-0.07, -0.04] ^**^ |
| Emotional Neglect | -0.09 [-0.10, -0.08] ^**^ | -0.07 [-0.09, -0.05] ^**^ | -0.10 [-0.11, -0.08] ^**^ |
|  | β-coefficients (95% CI) for z-standardized behavioral component score | | |
| CM events | Total sample | Men | Women |
| Physical Abuse | -0.11 [-0.12, -0.09] ^**^ | -0.10 [-0.12, -0.08] ^**^ | -0.12 [-0.13, -0.10] ^**^ |
| Sexual Abuse | -0.12 [-0.13, -0.10] ^**^ | -0.14 [-0.17, -0.11] ^**^ | -0.10 [-0.12, -0.08] ^**^ |
| Emotional Abuse | -0.11 [-0.12, -0.10] ^**^ | -0.11 [-0.13, -0.08] ^**^ | -0.11 [-0.12, -0.09] ^**^ |
| Physical Neglect | -0.03 [-0.05, -0.02] ^**^ | -0.03 [-0.05, -0.08] ^**^ | -0.04 [-0.05, -0.02] ^**^ |
| Emotional Neglect | -0.10 [-0.12, -0.09] ^**^ | -0.09 [-0.11, -0.07] ^**^ | -0.11 [-0.13, -0.09] ^**^ |
|  | β-coefficients (95% CI) for z-standardized health component score | | |
| CM events | Total sample | Men | Women |
| Physical Abuse | -0.09 [-0.10, -0.07] ^**^ | -0.08 [-0.10, -0.06] ^**^ | -0.09 [-0.11, -0.08] ^**^ |
| Sexual Abuse | -0.05 [-0.06, -0.03] ^**^ | -0.03 [-0.06, -0.002] ^**^ | -0.06 [-0.08, -0.04] ^**^ |
| Emotional Abuse | -0.05 [-0.06, -0.03] ^**^ | -0.04 [-0.06, -0.02] ^**^ | -0.05 [-0.07, -0.04] ^**^ |
| Physical Neglect | -0.06 [-0.07, -0.05] ^**^ | -0.07 [-0.09, -0.05] ^**^ | -0.05 [-0.07, -0.03] ^**^ |
| Emotional Neglect | -0.03 [-0.04, -0.02] ^**^ | -0.02 [-0.04, -0.002] ^**^ | -0.04 [-0.06, -0.03] ^**^ |
| ^*^p-value <0.05.  ^**^p-value <0.01.  Note: The line models for the total sample were adjusted for age, sex, ethnicity, education qualification, Townsend deprivation index, and Charlson Comorbidity Index. The sex-specific models were adjusted for age, ethnicity, education category, Townsend deprivation index, and Charlson Comorbidity Index.  Abbreviations: CI=confidence interval, CM=childhood maltreatment. | | | |

## Supplemental Table 11: Socio-demographic and health-related characteristics of participants included and excluded in the study

|  | Total population | Included participants | Excluded participants | p-value |
| --- | --- | --- | --- | --- |
| Age, years, Mean (±SD) | 56.5(±8.1) | 55.9 (±7.7) | 56.8 (±8.2) | <0.001 |
| Sex, n (%) |  |  |  | <0.001 |
| Female | 273,311 (54.4) | 89,036 (56.6) | 184,275 (53.4) |  |
| Male | 229,071 (45.6) | 68,217 (43.4) | 160,854 (46.6) |  |
| Ethnicity, n (%) |  |  |  | <0.001 |
| White | 472,586 (94.1) | 152,164 (96.8) | 320,422 (92.8) |  |
| Non-white | 28,897 (5.7) | 5,015 (3.1) | 23,882 (7.0) |  |
| Missing | 899 (0.2) | 74 (0.1) | 825 (0.2) |  |
| Education Qualification, n (%) |  |  |  | <0.001 |
| Low | 217,310 (43.3) | 47,865 (30.4) | 169,445 (49.1) |  |
| Medium | 81,104 (16.1) | 29,004 (18.4) | 52,100 (15.1) |  |
| High (College or University degree, NVQ or HND or HNC or equivalent) | 193,837 (38.6) | 78,886 (50.2) | 114,951 (33.3) |  |
| Missing | 10,131 (2.0) | 1,498 (1.0) | 8,633 (2.5) |  |
| Townsend Deprivation Index, n (%) |  |  |  | <0.001 |
| 1^st^ Quartile | 125,217 (24.9) | 44,213 (28.1) | 81,004 (23.5) |  |
| 2^nd^ Quartile | 124,814 (24.8) | 41,263 (26.2) | 83,551 (24.2) |  |
| 3^rd^ Quartile | 126,016 (25.1) | 39,719 (25.3) | 86,297 (25.0) |  |
| 4^th^ Quartile | 125,518 (25.0) | 31,849 (20.3) | 93,669 (27.1) |  |
| Missing | 817 (0.2) | 209 (0.1) | 608 (0.2) |  |
| LE8 score, **Mean (±SD)** | 63.6(**±12.8)** | 66.1(**±**12.3) | 62.4(**±12.8)** | <0.001 |
| Behavioral component score, **Mean (±SD)** | 65.4(**±16.4)** | 67.8(**±15.2)** | 64.3(**±16.8)** | <0.001 |
| Health component score, **Mean (±SD)** | 61.8(**±17.9)** | 64.5(**±17.5)** | 60.5(**±17.9)** | <0.001 |
| LE8 score, n (%) |  |  |  | <0.001 |
| Low | 71,246 (14.2) | 15,202 (9.7) | 56,044 (16.2) |  |
| Moderate | 376,804 (75.0) | 120,112 (76.4) | 256,692 (74.4) |  |
| High | 52,260 (10.4) | 21,790 (13.8) | 30,470 (8.8) |  |
| Missing | 2,072 (0.4) | 149 (0.1) | 1,923 (0.6) |  |
| Charlson Comorbidity Index, n (%) |  |  |  | <0.001 |
| None | 438,109 (87.2) | 142,418 (90.6) | 295,691 (85.7) |  |
| Mild | 54,125 (10.8) | 12,902 (8.2) | 41,223 (11.9) |  |
| Moderate | 6,021 (1.2) | 1,130 (0.7) | 4,891 (1.4) |  |
| Severe | 4,127 (0.8) | 803 (0.5) | 3,324 (1.0) |  |

Abbreviations: SD=standard deviation, LE8=Life’s Essential 8.

## Supplemental Table 12: Association between LE8 scores and CVD risk

| CVD type | Hazard Ratio (95% CI) per 1 unit increase in LE8 score | | |
| --- | --- | --- | --- |
|  | Total | Men | Women |
| Myocardial Infarction | 0.97 [0.97-0.98] ^**^ | 0.98 [0.97-0.98] ^**^ | 0.97 [0.96-0.97] ^**^ |
| Cerebrovascular Disease | 0.98 [0.98-0.99] ^**^ | 0.98 [0.98-0.99] ^**^ | 0.98 [0.98-0.99] ^**^ |
| Congestive Heart Failure | 0.97 [0.97-0.98] ^**^ | 0.98 [0.97-0.98] ^**^ | 0.97 [0.96-0.98] ^**^ |
| Peripheral Vascular Disease | 0.97 [0.97-0.98] ^**^ | 0.97 [0.97-0.98] ^**^ | 0.98 [0.97-0.98] ^**^ |

^**^ p-value <0.01.

Note: Cox proportional hazard regression models for the total sample were adjusted for age, sex, ethnicity, education qualification, Townsend deprivation index, childhood maltreatment, and Charlson Comorbidity Index, while sex-specific models were adjusted for all except sex.

Abbreviations: CI=confidence interval, CVD= cardiovascular diseas

## Supplemental Table 13: Association between LE8 score and All-Cause Mortality

| Models | No. of CM | Hazard Ratio (95% CI) for all-cause mortality per 1 unit increase in LE8 score | | |
| --- | --- | --- | --- | --- |
|  |  | Total | Men | Women |
| Model 1 |  | 0.98 [0.97-0.98] ^**^ | 0.98 [0.97-0.98] ^**^ | 0.98 [0.97-0.98] ^**^ |
| Mode 2 | No CM | 0.98 [0.97-0.99] ^**^ | 0.98 [0.97-0.98] ^**^ | 0.98 [0.98-0.99] ^**^ |
|  | 1⎯2 CM | 0.98 [0.98-0.99] ^**^ | 0.98 [0.97-0.99] ^**^ | 0.98 [0.98-0.99] ^**^ |
|  | ≥3 CM | 0.97 [0.96-0.98] ^**^ | 0.97 [0.96-0.98] ^**^ | 0.98 [0.97-0.99] ^**^ |

^**^ p-value <0.01.

Note:

Model 1: Cox proportional hazard regression models for the total sample were adjusted for age, sex, ethnicity, education qualification, Townsend deprivation index, childhood maltreatment, and Charlson Comorbidity Index, while sex-specific models were adjusted for all except sex.

Model 2: Cox proportional hazard regression models stratified by childhood maltreatment. Models for the total sample were adjusted for age, sex, ethnicity, education qualification, Townsend deprivation index, and Charlson Comorbidity Index, while sex-specific models were adjusted for all except sex.

Abbreviations: CI=confidence interval, CM= Childhood maltreatment


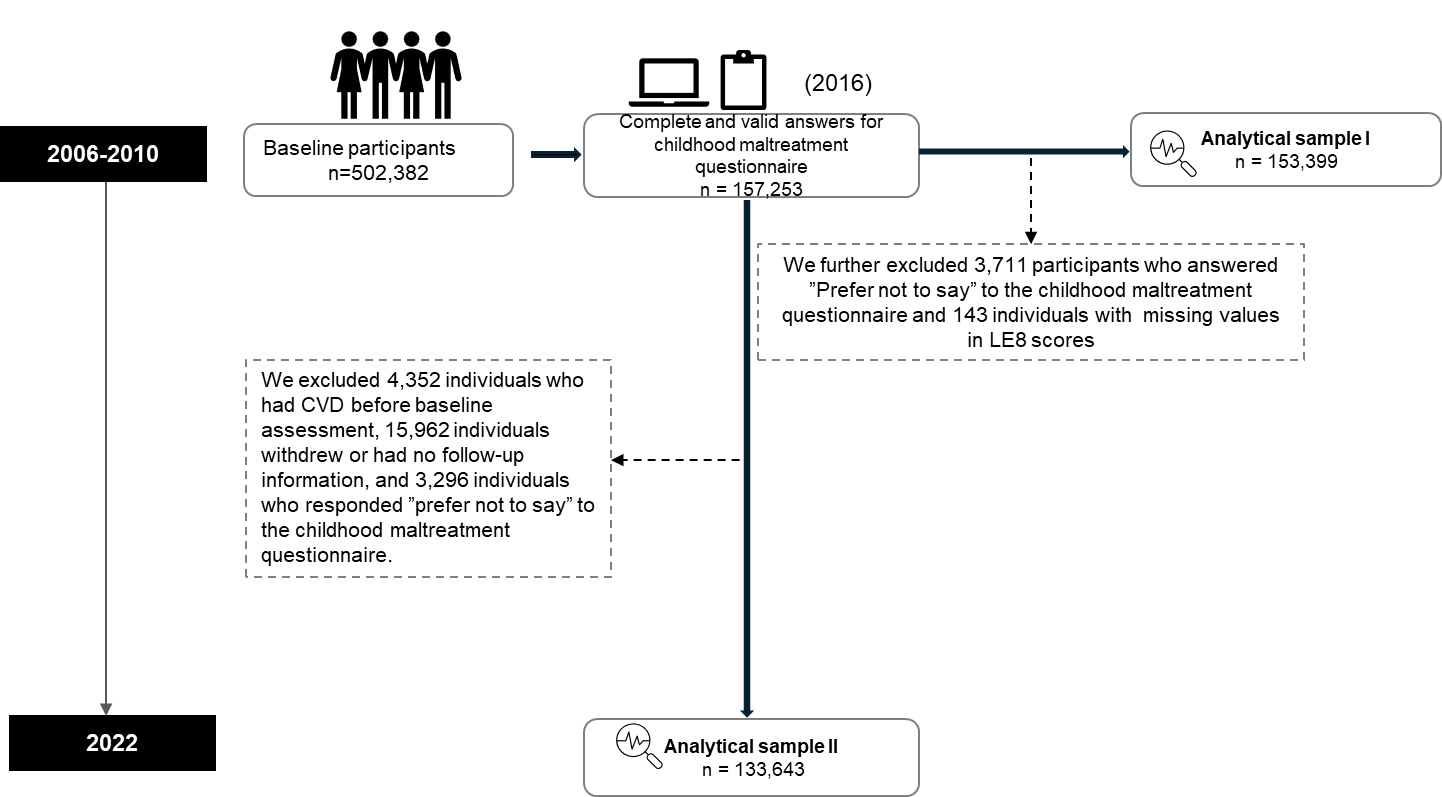


## Supplemental Figure 1: Flow-chart of study participants in the UK Biobank


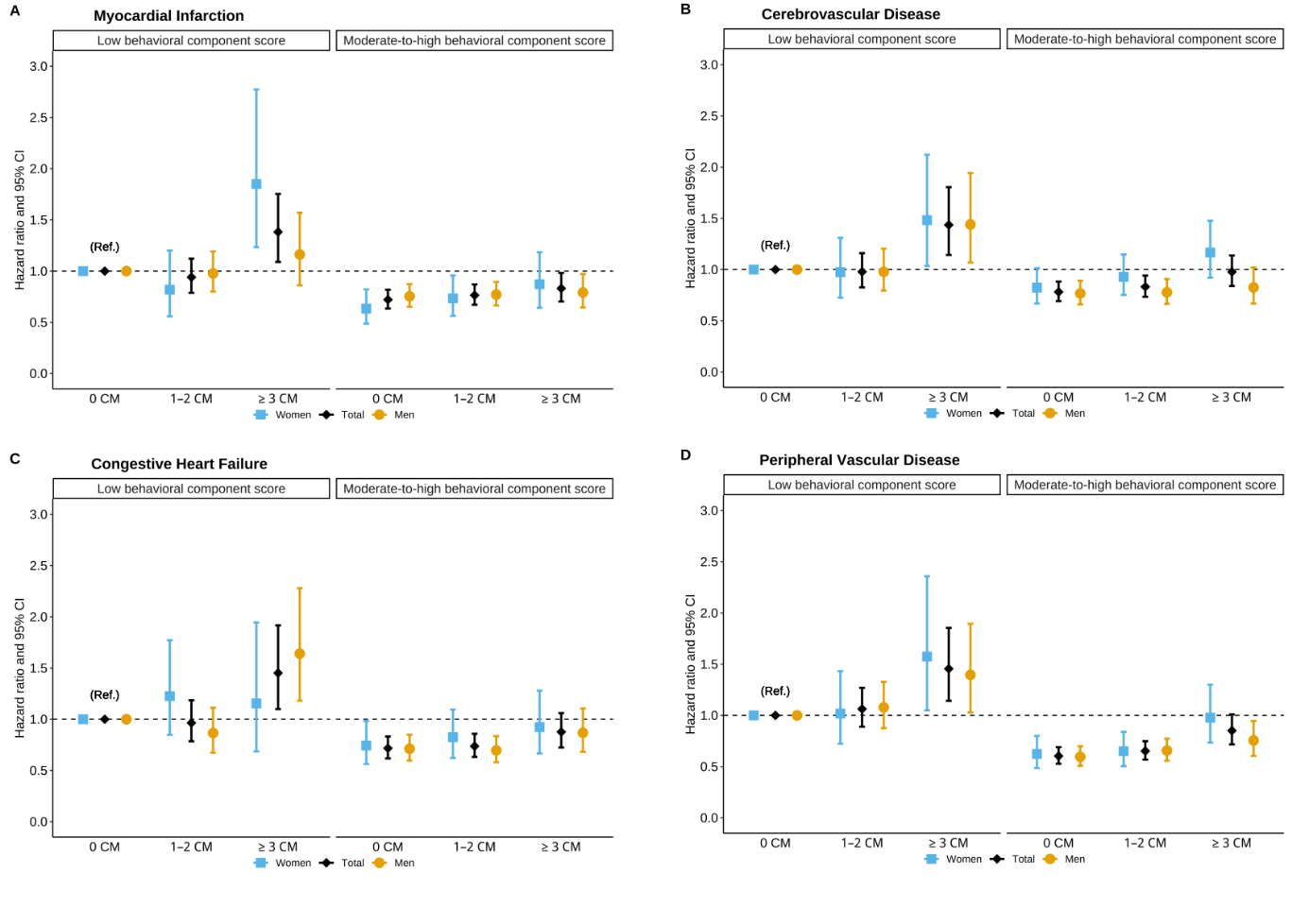


## Supplemental Figure 2: Association between childhood maltreatment and incident CVD events by behavioral component levels and sex

Note: Models for the total sample were adjusted for age, sex, ethnicity, education qualification, TDI, and CCI, while sex-specific models were adjusted for all except sex.

Abbreviations: CI= confidence interval, CM=childhood maltreatment


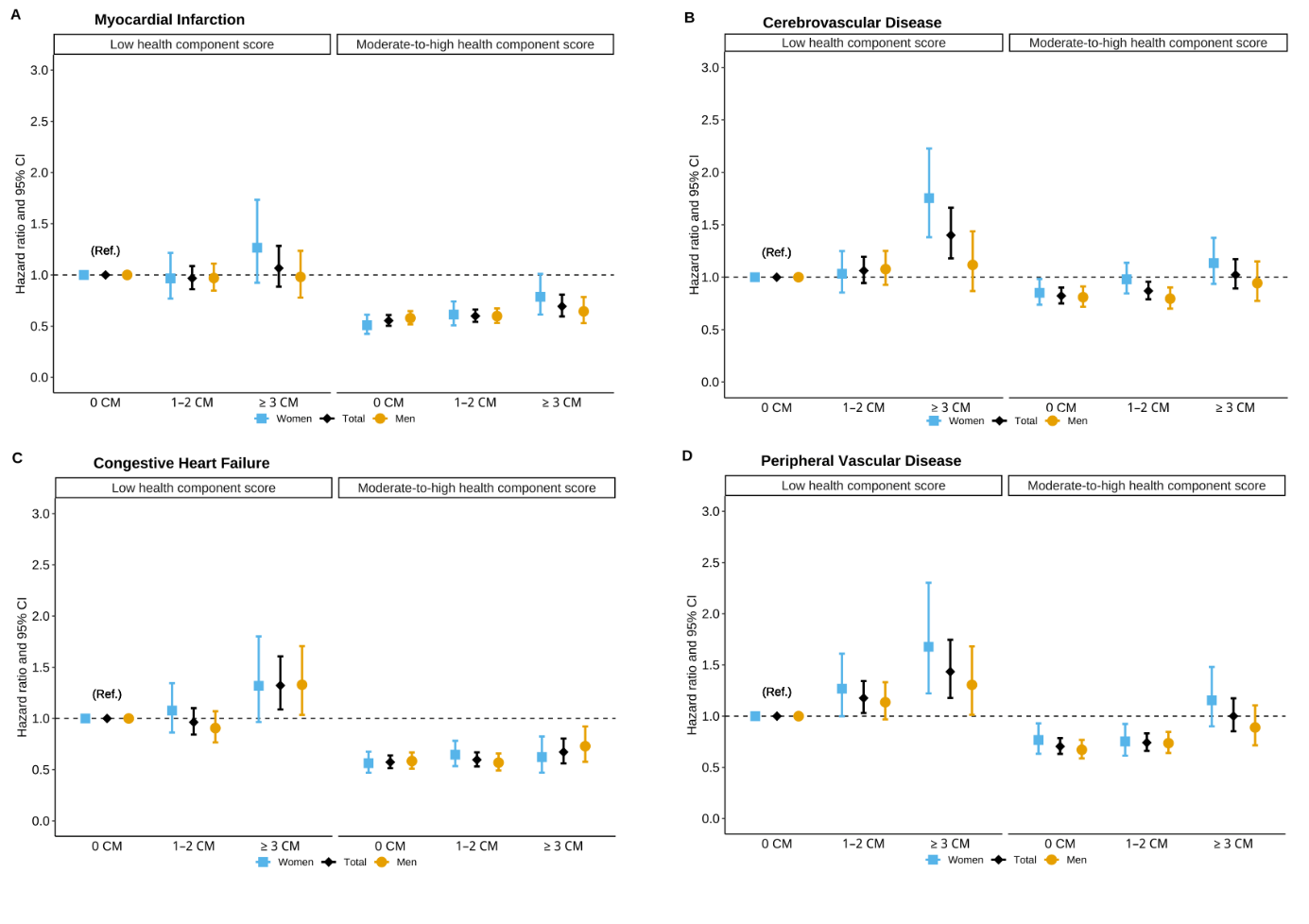


## Supplemental Figure 3: Association between childhood maltreatment and incident CVD events by health component and sex

Note: Models for the total sample were adjusted for age, sex, ethnicity, education qualification, TDI, and CCI, while sex-specific models were adjusted for all except sex.

Abbreviations: CI= confidence interval; CM=childhood maltreatment


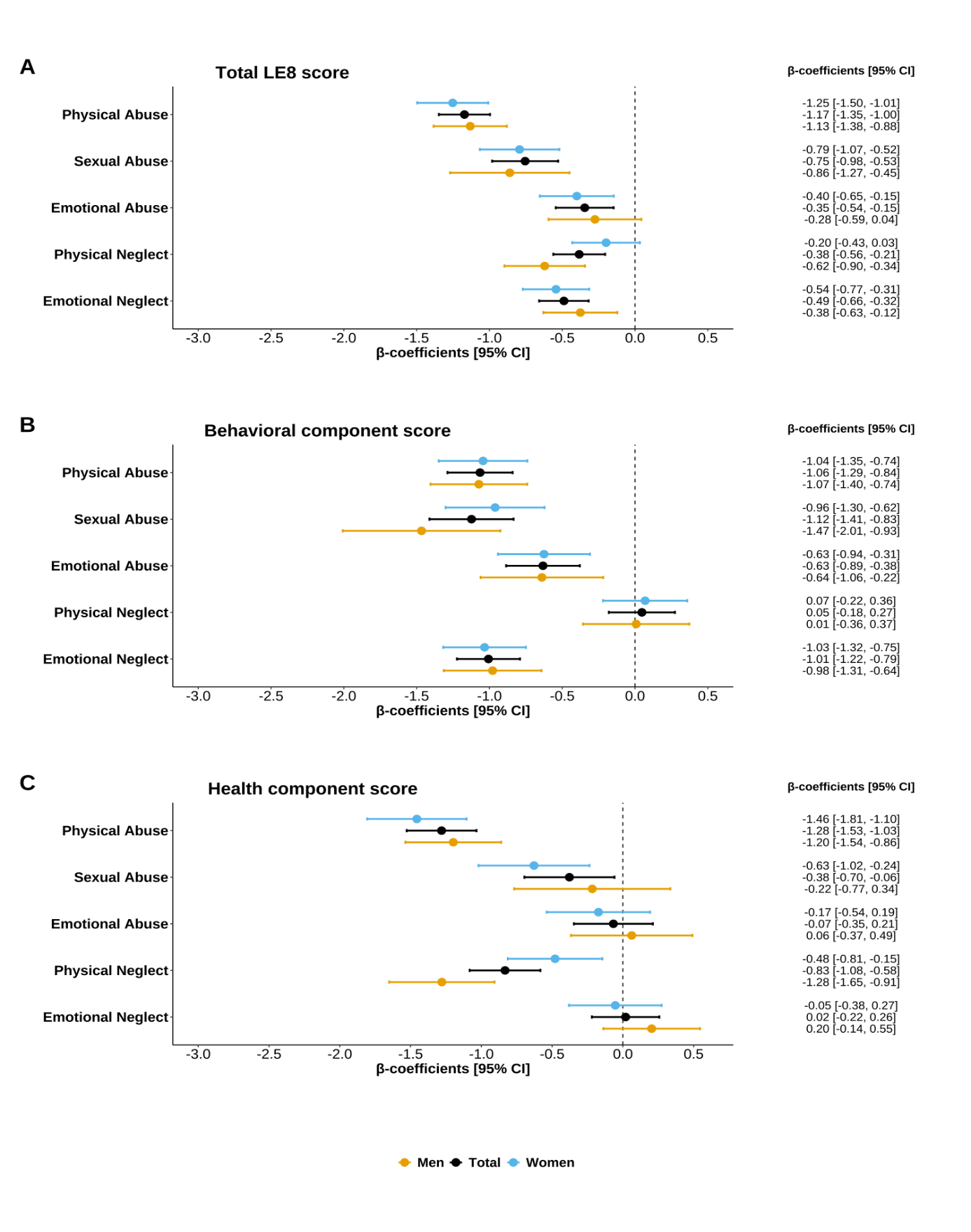


## Supplemental Figure 4: β-coefficients of LE8 scores in relation to specific childhood maltreatment events

*Note*. All linear regression models in the total sample were adjusted for each maltreatment event, age, sex, ethnicity, education qualification, Townsend Deprivation Index, and Charlson Comorbidity Index, while sex-specific models were adjusted for all except sex.

Abbreviations: CI=confidence interval, LE8=Life’s Essential 8.


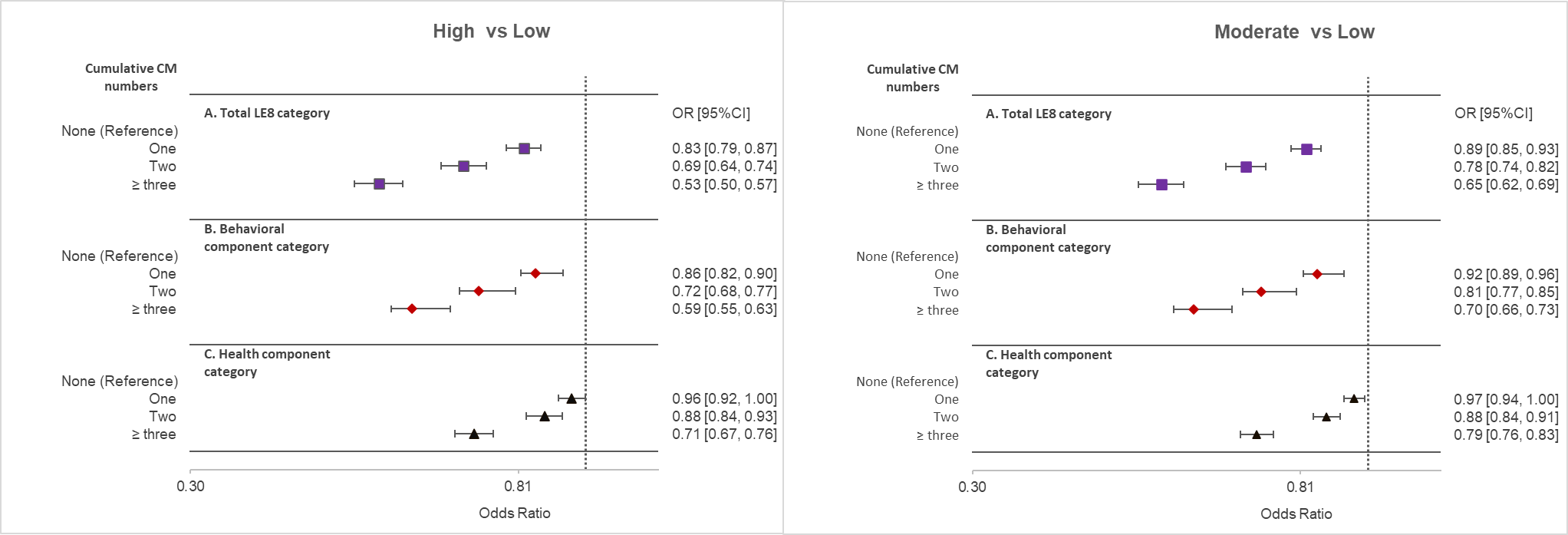


## Supplemental Figure 5: Association between cumulative childhood maltreatment numbers and categories of LE8

Note. All models in the total sample were adjusted for age, sex, ethnicity, education qualification, Townsend Deprivation Index, and Charlson Comorbidity Index.

Abbreviations: CI=confidence interval, CM: childhood maltreatment, LE8=Life’s Essential 8, OR=odds ratio.


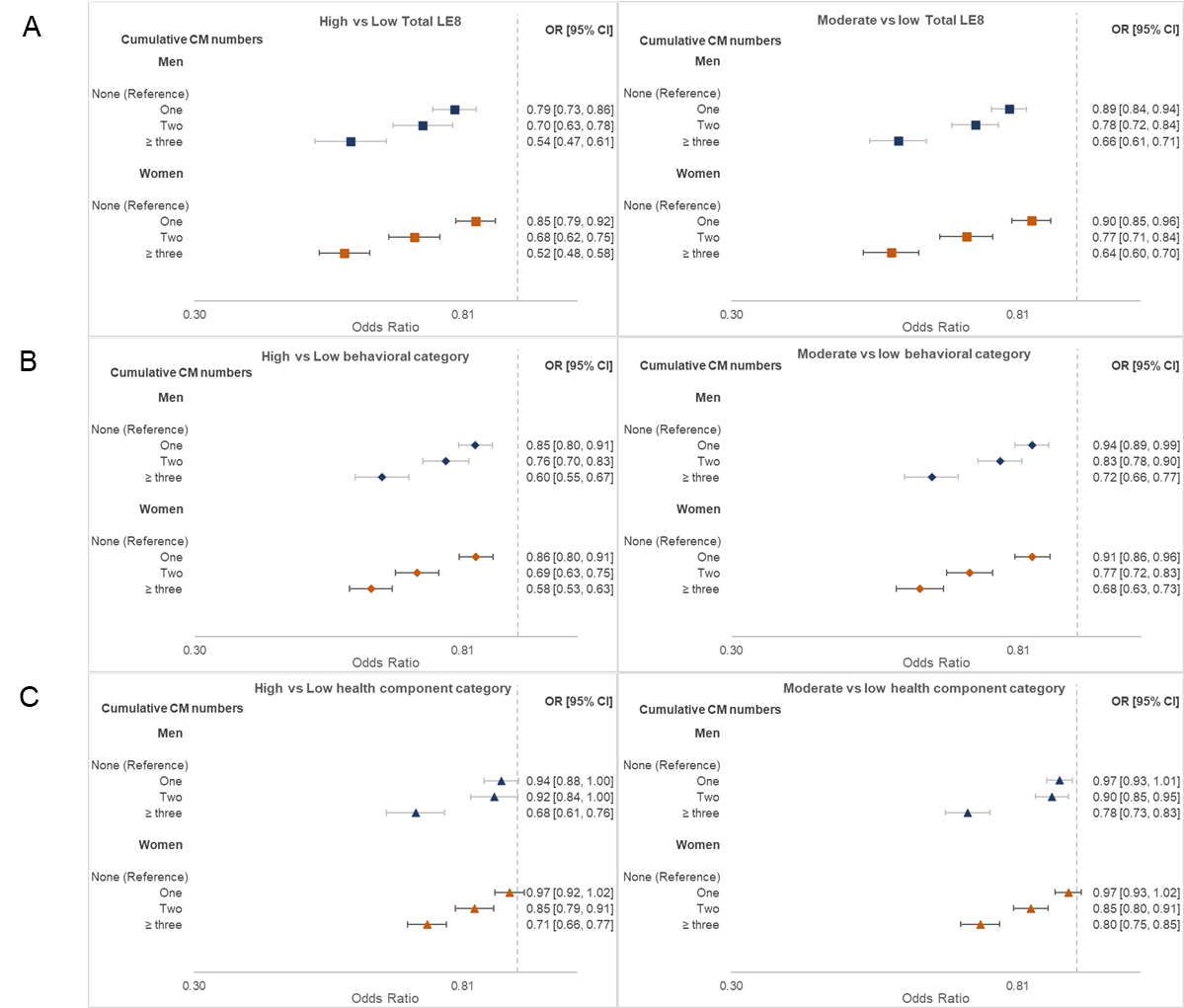


## Supplemental Figure 6: Association between cumulative childhood maltreatment numbers and LE8 categories by sex

Note. All models in the total sample were adjusted for age, ethnicity, education qualification, Townsend Deprivation Index, and Charlson Comorbidity Index.

Abbreviations: CI=confidence interval, CM=childhood maltreatment, LE8=Life’s Essential 8, OR=odds ratio.

## References

1. Lloyd-Jones DM, Allen NB, Anderson CAM, et al. Life’s Essential 8: Updating and Enhancing the American Heart Association’s Construct of Cardiovascular Health: A Presidential Advisory From the American Heart Association. *Circulation*. 2022;146(5). doi:10.1161/CIR.0000000000001078

2. Beydoun HA, Beydoun MA, Meirelles O, et al. Cardiovascular health, infection burden, and incident dementia in the UK Biobank. *Alzheimer’s & Dementia*. 2023;19(10):4475-4487. doi:10.1002/alz.13405

3. Hu HY, Ma YH, Deng YT, et al. Residential greenness and risk of incident dementia: A prospective study of 375,342 participants. *Environ Res*. 2023;216(Pt 3):114703. doi:10.1016/j.envres.2022.114703
